# Logistic regression analysis of populations of electrophysiological models to assess proarrythmic risk

**DOI:** 10.1016/j.mex.2016.12.002

**Published:** 2016-12-23

**Authors:** Stefano Morotti, Eleonora Grandi

**Affiliations:** Department of Pharmacology, University of California Davis, Davis, CA, USA

**Keywords:** Logistic regression analysis of populations of electrophysiological models, Mathematical modeling, Cardiac arrhythmia, Sensitivity analysis, Logistic regression

## Abstract

Population-based computational approaches have been developed in recent years and helped to gain insight into arrhythmia mechanisms, and intra- and inter-patient variability (e.g., in drug responses). Here, we illustrate the use of multivariable logistic regression to analyze the factors that enhance or reduce the susceptibility to cellular arrhythmogenic events. As an example, we generate 1000 model variants by randomly modifying ionic conductances and maximal rates of ion transports in our atrial myocyte model and simulate an arrhythmia-provoking protocol that enhances early afterdepolarization (EAD) proclivity. We then treat EAD occurrence as a categorical, yes or no variable, and perform logistic regression to relate perturbations in model parameters to the presence/absence of EADs. We find that EAD formation is sensitive to the conductance of the voltage-gated Na^+^, the acetylcholine-sensitive and ultra-rapid K^+^ channels, and the Na^+^/Ca^2+^ exchange current, which matches our mechanistic understanding of the process and preliminary sensitivity analysis.

The described technique:

• allows investigating the factors underlying dichotomous outcomes, and is therefore a useful tool improve our understanding of arrhythmic risk;
• is valid for analyzing both deterministic and stochastic models, and various phenomena (e.g., delayed afterdepolarizations and Ca^2+^ sparks);
• is computationally more efficient than one-at-a-time parameter sensitivity analysis.

## Graphical abstract

**Figure:**
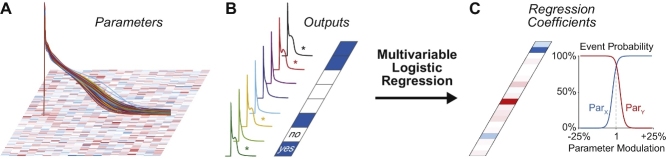

## Method details

Population-based approaches have been widely adopted in the recent years in the field of computational cardiac electrophysiology [1], [2], [3], [4], [5], [6], [7], [8]. The classical analysis performed with a single action potential (AP) model has been extended to the study of the properties emerging in a broad group of models (called “population”). Each individual in the population is obtained from the same parent model (called “baseline”) by randomly perturbing several parameters. These differences in model parameterization reflect the natural variability among cells in a tissue (intra-subject variability) or among different individuals (inter-subject variability). Thus, by investigating a more realistic and comprehensive scenario, this approach allows obtaining new insights in several aspects of myocyte function in health and disease, including arrhythmia mechanisms and responses to drugs [9]. Moreover, the assessment of sensitivity of model predictions to parameter perturbations ensures enhanced robustness of the results of computational studies [10]. However, the choice of appropriate mathematical and statistical tools is fundamental when performing sensitivity analysis on a population of hundreds or thousands AP models. Here, we illustrate the use of multivariable logistic regression to analyze the factors underlying the occurrence of a dichotomous outcome (such as the development of cellular arrhythmogenic events).

### Explanation of the methodology

Regression analysis is a statistical process used to estimate relationships among variables in a system [11], [12]. It is usually applied to correlate modifications in independent variables (called “predictors”) to the consequent effect on dependent variables. The result of regression analysis is a function of the independent variables (called “regression function”), which is used to predict the values of the dependent variables (e.g., to estimate a certain outcome after changes in fixed system properties). More specifically, the result of this analysis is the set of regression coefficients used in the regression function. The general principles of regression analysis are formalized in the following equation:

**X** * **B** = **Y**^∼^ ≈ **Y**

where **X** and **Y** are the matrices of the independent and dependent variables, respectively. Based on **X** and **Y**, this technique allows the determination of the regression coefficients (in the matrix **B**), which can be used to estimate **Y** (**Y^∼^**) given **X**.

In the field of computational cardiac electrophysiology, sensitivity analysis of population of AP models is usually performed to assess how variations in model parameters (e.g., conductances of ion channels) affect model outputs, such as AP duration (APD) or Ca^2+^ transient amplitude. In this case, when the outputs of interest are continuous variables, linear multivariable regression analysis can be performed, and the resulting regression function allows predicting how these outputs change (in a continuous range) upon modifications in model parameters [1]. In a population of *n_m_* AP models, obtained by varying *n_p_* parameters in a baseline model, the size of the matrix of the predictors **X** is *n_m_ x n_p_* (*rows x columns*). The matrix **Y** reports different outputs (*n_o_*) identified in each model of the population (size *n_m_ x n_o_*). In general, the matrix **B** contains a coefficient describing the effect of perturbation in each parameter on each output (size *n_p_ x n_o_*).

A different statistical process, the multivariable logistic regression, can be used instead when the outputs of interest are described as Boolean variables (i.e., yes/no, true/false, presence/absence) [2], [8]. This analysis allows estimating how perturbations in model parameters affect the probability that a certain dichotomous outcome will occur. A typical example (described in the next section) is the occurrence of cellular arrhythmogenic events such as early afterdepolarizations (EADs). The regression function used here is the logistic curve, an S-shape (sigmoidal) curve with values between 0 and 1. These extremes represent, respectively, low and high probabilities of occurrence of a certain event.

**Fig. 1:**
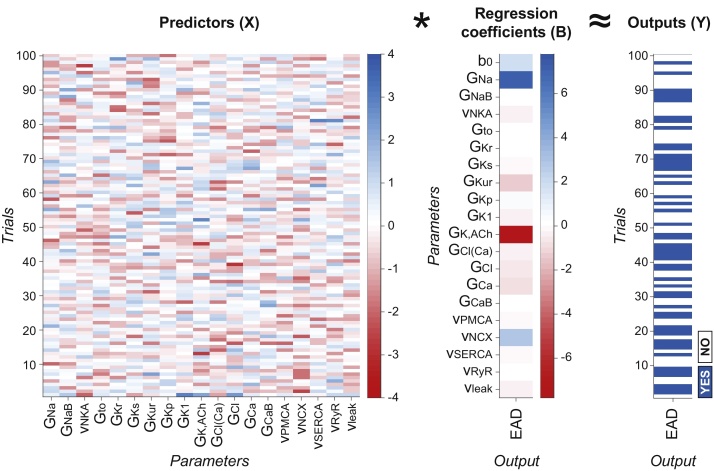
Schematic representation of predictor, coefficient, and output matrices in logistic regression analysis. In the example presented in this paper, the matrix of the predictors **X** contains information about the scale factors used to generate a population of 1000 AP models (only 100 cases are shown here for the sake of simplicity) by perturbing the baseline value of 19 parameters (defined in Table 1). The same proarrhythmic protocol is simulated with all the models in the population, and the presence of EADs is assessed in each case, thereby obtaining the binary elements of the matrix of the outputs **Y**. The result of logistic regression analysis is the matrix of the coefficients **B**, used to estimate the effect of perturbations in model parameters on the probability of arrhythmogenesis. Here both **Y** and **B** are arrays because only one output (i.e., EAD development) is evaluated in this example. Values in **X** (obtained from the scale factors via log-transformation and conversion into z-scores, see text) and **B** (regression coefficients, shown also in Fig. 4) are dimensionless quantities, while elements of **Y** are Boolean variables.

Logistic regression analysis correlates perturbations to model parameters (used to determine the matrix of the predictors **X** as described below) to the values in **Y**, which consist of binary variables corresponding to whether or not the outcome of interest has occurred. The resulting matrix **B** contains a set of *n_p_* *+* *1* regression coefficients (array **b**) for each output studied (Fig. 1). Each set includes *n_p_* coefficients (b_1_-b_np_) corresponding to the effect induced by perturbations in the *n_p_* parameters, and an additional coefficient b_0_ (called “intercept term”). In the generic model configuration (described by the array **x**), values in **b** are used to assess the probability P of occurrence of the desired output as follows:

P(**x**, **b**) = 1/(1 + exp (–(b_0_ + x_1_*b_1_ + x_2_*b_2_ + … + x_np_*b_np_)))

In the next section we describe how logistic regression is applied to the study of EAD formation and how its results can be interpreted. We will also explain how this analysis is implemented with MATLAB (The MathWorks, Natick, MA, USA). Our codes, generated with the MATLAB version R2016a, are freely available for download at the following webpages: somapp.ucdmc.ucdavis.edu/Pharmacology/bers & elegrandi.wixsite.com/grandilab/downloads.

### Example of application

An application of the described methodology is the evaluation of the factors influencing arrhythmia proclivity. In the example presented here, we apply logistic regression to study the mechanisms responsible for the development of EADs, treated as a Boolean variable.

In a recent computational study [13], we used our established model of AP and Ca^2+^ handling in the human atrial myocyte [14] to describe a novel mechanism for phase-3 EADs. In canine atrial preparations, Burashnikov and Antzelevitch have shown that phase-3 EADs arise after termination of atrial fibrillation (AF), and are the trigger for re-induction of fibrillation [15]. Analogous triggered phenomena have been described in canine pulmonary veins with autonomic nerve stimulation [16], or combined acetylcholine (ACh) and epinephrine challenge [17]. These protocols mimic simultaneous sympathovagal discharges that commonly precede AF onset [18], [19], [20]. Experimental studies have shown that initiation of these EADs occurs secondary to APD shortening by ACh (via ACh-sensitive K^+^ current, I_K,ACh_), potentiated sarcoplasmic reticulum (SR) Ca^2+^ release, and enhanced Na^+^/Ca^2+^ exchange (NCX) [16]. We demonstrated that these allow for cardiac Na^+^ current (I_Na_) recovery and non-equilibrium reactivation, which ultimately drives the EADs [13]. Based on data from models of acute AF, and data describing the early electrophysiological changes in AF [21], we speculated that EADs are a likely source of ectopy early in disease progression (e.g., paroxysmal AF), particularly in the pulmonary veins, where the short AP and late AP plateau (at negative membrane potential) induced by autonomic challenge can facilitate non-equilibrium I_Na_ reactivation. The same phenomenon was previously observed in mouse ventricular myocytes [22].

The described arrhythmogenic process involves many elements that are important regulators of myocyte electrophysiology and Ca^2+^ handling (such as I_Na_, NCX and SERCA activity, and cholinergic and adrenergic signaling cascades). Thus, the use of logistic regression analysis is particularly useful in this context to assess the impact of each ion current or transporter on arrhythmia mechanisms, thereby providing potentially useful insight for the identification of therapeutic targets.

**Fig. 2:**
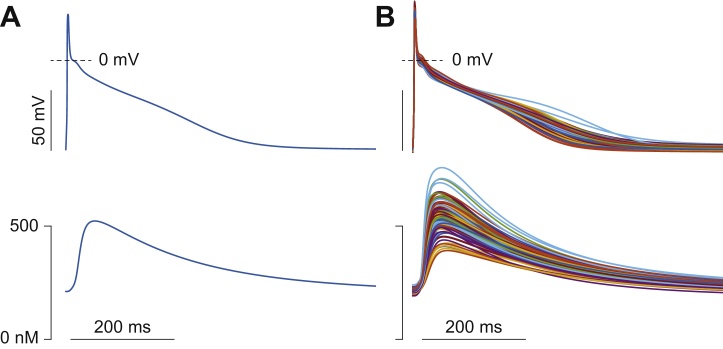
Populations of AP models. AP (top panels) and Ca^2+^ transient (bottom) evoked stimulating at 1 Hz the baseline model (A) and 100 variants randomly selected in our population (B).

Here we use our published model [13] as a baseline to build a population of 1000 elements (*n_m_* = 1000) by randomly modifying 19 parameters (*n_p_* = 19) corresponding to conductances (G) of ion currents and maximal rates (v) of ion transports (see parameter definition in Table 1). We follow the approach described by Sobie [1]: for each individual, we modify the baseline value of each parameter by multiplication for a log-normally distributed random scale factor k. We obtain these factors using the following MATLAB command:

k = exp(σ*randn)

where *randn* is a function that generates normally distributed pseudorandom numbers, and σ is the parameter that determines the degree of variability. More specifically, σ is the standard deviation of the set of scale factors k generated for each parameter (around the mean value μ = 1). We use here the same value of σ (0.1) for all the parameters. Alternatively, different standard deviations can be used to reproduce the degree of variability experimentally determined for each parameter [6], [7], [23]. Note that while this choice does not alter the subsequent steps of this analysis, when increasing the range of variability of the model parameter, one needs to *(i)* discard non-physiological models; and *(ii)* ensure representative coverage of the parameter space (convergence of sensitivity coefficients upon stable values) by increasing the number of model variants [2]. All scale factors are allocated into the *1000* *×* *19* matrix **K**, and used to simulate the different models in our population. Because of the relatively small σ chosen here for simplicity, APs and Ca^2+^ transients in our population fall within the normal range of experimental variability (Fig. 2), and sensitivity coefficients converge upon stable values with population sizes as small as 100 variants (not shown, see also [4]).

**Fig. 3:**
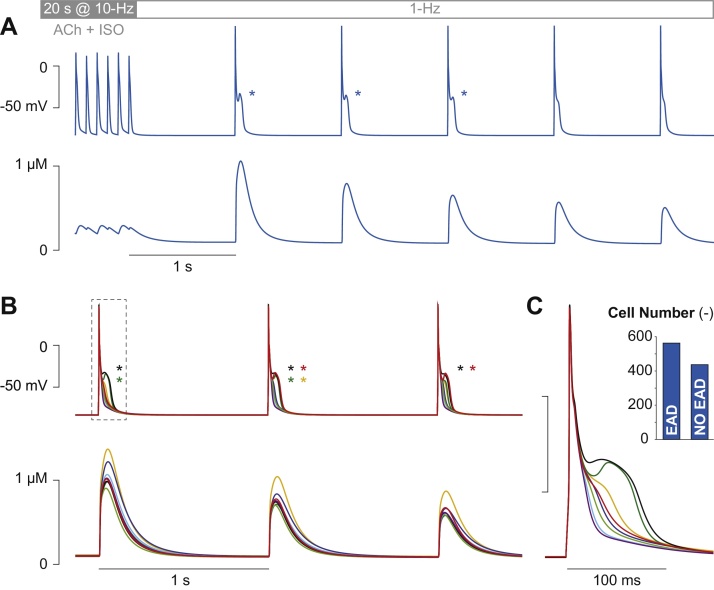
Arrhythmia-provoking protocol. Membrane potential (top panels) and Ca^2+^ concentration (bottom) evoked with the baseline model (A) and 8 randomly selected variants in our population (B). EAD presence is indicated by asterisks. Panel A shows the last 6 beats of the 20-s phase at 10-Hz pacing, followed by 5 beats at normal rate (1 Hz). The first 3 beats at 1 Hz are reported in B. The first APs obtained after the pause is shown in panel C to highlight the variability among the different responses. The inset in C reports the number of simulations (i.e., models) in which at least one EAD is evoked during the 5 beats at 1-Hz pacing.

We repeat the proarrhythmic protocol described in [13] with our 1000 AP model variants to evaluate the occurrence of EADs in each simulation. We start all the simulations from the steady-state conditions obtained by pacing the baseline model at 1 Hz in the presence of ACh (0.1 μM). Then, we simulate instantaneous administration of isoproterenol (ISO, 1 μM) at the beginning of a 20-s phase at 10-Hz pacing, followed by 5 s at 1 Hz to mimic spontaneous termination of tachyarrhythmia (Fig. 3A). Presence of EADs is assessed during the 5 beats at 1-Hz pacing, thereby creating the array **y** with 1000 Boolean variables (0 for absence and 1 for occurrence of at least one EAD). EADs are detected as positive deflections of the membrane potential derivative during AP repolarization.

We perform logistic regression using the MATLAB command *mnrfit*, which fits a nominal multinomial logistic regression model for the predictor matrix **X** and the output matrix **Y** (Fig. 1):

**B** = *mnrfit*(**X**, **Y**)

The matrix **X** is here derived from the matrix **K** containing the scale factors. First, **K** is log-transformed to obtain the matrix **K_L_**:

**K_L_** = log(**K**)

In our case, since each column in **K** (i.e., 1000 scale factors for the same parameter) has μ = 1 and σ = 0.1, each column in **K_L_** has μ = 0 and σ = 0.1. To obtain **X**, **K_L_** is converted into z-scores by subtracting the mean across all models and dividing by the standard deviation. More specifically, we perform the following operation for each column:

**x** = (**k_L_** − μ_kL_)/σ_kL_

where **k_L_** and **x** are generic columns in **K_L_** and **X**, and μ_kL_ and σ_kL_ are mean and standard deviation computed on the column **k_L_**, respectively. Therefore, the scale factor k corresponding to x, a generic element of **X**, can be determined with the following formulation:

k = exp(σ_kL_ × x−μ_kL_)

The resulting matrix of the predictors **X** has the same size of **K** (*1000* *×* *19*) and μ = 0 and σ = 1 for each column, independently of the properties of **K**, which facilitates interpretation of the results, as described below. In fact, **K** may contain either the scale factors (as in this example, where each column has μ = 1) or the actual parameter values used in each model of the population (where the mean of each column corresponds to the parameter value in the baseline model). The degree of variation can also be different for each parameter. Since each column in **K** may have different μ and σ values, log-transformation and following conversion into z-scores ensure that each column in **X** has same μ and σ (0 and 1, respectively). Since here we are interested in only one output (*n_o_* = 1), the matrix **Y** is reduced to one column (*1* *×* *1000*), containing the binary information about presence/absence of EADs. In this case, the input of *mnrfit* must contain positive integer category numbers, thus we modify the array **y** to indicate the presence of EADs with 1 and absence of EADs with 2 (rather than 0). Also **B**, the output of *mnrfit*, consists of only one column (hence called array **b**) of size *1* *×* *20*, containing the intercept term b_0_ and the 19 coefficients corresponding to the perturbed parameters.

When simulating the proarrhythmic protocol with our baseline model, we obtained EADs in the first 3 beats in the 1-Hz phase (Fig. 3A, see asterisks) [13]. Different behaviors are observed within the population, as shown in Fig. 3B and C. The overall analysis shows that our protocol induced EADs in 563 out of 1000 model variants (Fig. 3C, inset). In this sub-population of EAD-positive cells, only one episode (see yellow example in Fig. 3B) or multiple EADs (black example) are predicted. In 18 simulations, a delayed afterdepolarization develops between the last beat at 10-Hz and the first stimulated beat at 1-Hz (not shown). In these cases, included in the analysis, EADs are observed in 9 out of 18 models.

**Fig. 4:**
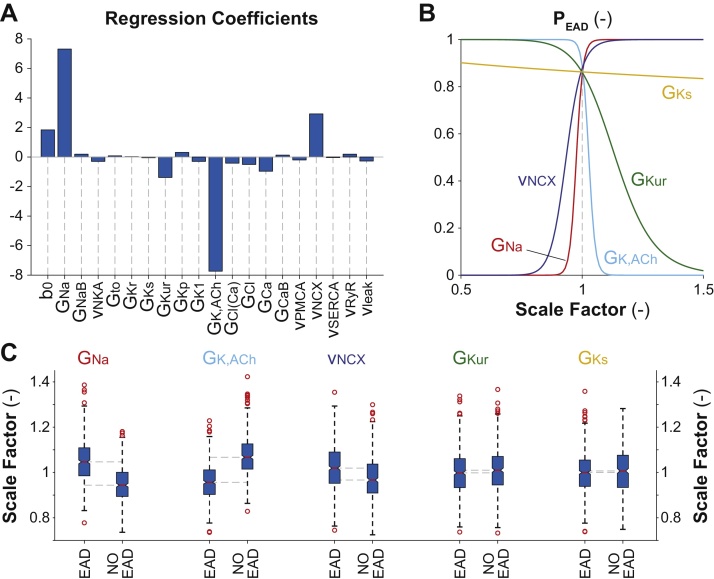
Results of logistic regression analysis. A) Coefficients of the multinomial logistic regression model describing the probability of EAD development in the models of our population. B) Effects on P_EAD_ induced by modulation of one model parameter at the time (G_Na_, G_K,ACh_, v_NCX_, G_Kur_ and G_Ks_). C) Statistical analysis on the values of the perturbations to these 5 parameters in the two sub-populations of AP models with or without EADs performed with the MATLAB command *boxplot*. The central red line indicates the median of each group (reported in Table 2), while the blue box represents the central 50% of the data. The lower and upper boundaries of the central box are, respectively, at the 25th and 75th percentiles. The dotted vertical lines (called “whiskers”) maximally extend to 1.5 times the height (called “inter-quartile range”) of the central box. Individual values outside the range of the whiskers are considered outliers (shown here with red circles). The extremes of the lateral notches of the central box are determined as q_50_ ±1.57(q_75_–q_25_)/sqrt(n), where q_25_, q_50_ and q_75_ are the 25th, 50th (i.e., the median) and 75th percentiles, respectively, and n is the number of observations in each group. These values (reported in Table 2) mark the 95% confidence interval for the medians (i.e., if the notches from two boxplots do not overlap, one can assume that the medians are different with a significance level of 0.05).

The 20 coefficients obtained with the logistic regression analysis are reported in Fig. 4A. The use of **X** (rather than **K**) allows easily interpreting these coefficients to assess changes in the probability that the cell will develop EADs (P_EAD_). Except for b_0_, the values of all the other regression coefficients reflect the effect of 1-standard deviation increase from the (log-transformed) baseline value of the corresponding model parameter. Hence, all these coefficients can be directly compared, even if the related parameters are characterized by different variability ranges around different mean values [2]. Augmenting the parameters associated with positive coefficients increases P_EAD_. This probability increases also when reducing the parameters associated with negative coefficients. The largest bars correspond to model parameters that mostly influence the development of EADs, namely G_Na_, G_K,ACh_, v_NCX_, and G_Kur_. In Fig. 4B we quantify the effect of modulation in these parameters (and in G_Ks_) on P_EAD_. These traces are obtained by assuming changes in one parameter at the time (with respect to the baseline value), using the following equation:

P_EAD_(x, b) = 1/(1 + exp (–(b_0_ + x*b)))

where b is the regression coefficient corresponding to the perturbed parameter, and x is the degree of variation (log-transformed and converted into z-score). Here we show the effects of ±50% modulation in these model parameters (i.e., corresponding to variations of the scale factors between 0.5 and 1.5). Note that, given a scale factor for a certain parameter, the corresponding x is obtained using the values of μ_kL_ and σ_kL_ estimated for the same parameter in the population, as described before. In absence of perturbations (i.e., when x = 0), P_EAD_ depends only on b_0_. In this case, which corresponds also to the probability calculated for the baseline model (where the array **x** is null), P_EAD_ is high (∼0.86). Indeed, we obtain EADs in this condition (Fig. 3A).

As described in our previous paper, these phase-3 EADs are a consequence of non-equilibrium reactivation of I_Na_, and their occurrence can be prevented by blocking I_Na_ with ranolazine [13]. In our preliminary sensitivity analysis (see Supplementary data in [13]) we also showed that the same result can be obtained with 5% reduction in G_Na_, but not with ±5% modulation in other model parameters. Thus, finding that G_Na_ is the most important parameter (largest bar in Fig. 4A, and steepest relationship in Fig. 4B) is not unexpected. Fig. 4B shows that also changes in G_K,ACh_ and v_NCX_ have important and opposite effects on P_EAD_: increased G_K,ACh_ (negative coefficient) and decreased NCX activity (positive coefficient) are protective. The involvement of NCX is also to be expected. Indeed, our proposed mechanism involves I_Na_ reactivation, which is favored by NCX-mediated AP plateau prolongation. NCX enhancement is induced by Ca^2+^ augmentation upon re-establishment of normal pacing frequency. Decreased v_NCX_ shortens the AP, thereby hindering I_Na_ reactivation. The same effect is also obtained when the repolarizing ACh-sensitive and ultra-rapid K^+^ currents are enhanced (i.e., increased G_K,ACh_ and G_Kur_). Thus, while I_K,ACh_-induced APD shortening is required to hasten I_Na_ recovery from inactivation, excessive I_K,ACh_ prevents EADs by shifting the balance between repolarizing and depolarizing currents. Indeed, we had previously observed that increasing [ACh] prevents EADs in the model, primarily by increasing the repolarization rate [13].

We compare the scale factor values relative to these parameters in the two EAD-positive and EAD-negative sub-populations. Fig. 4C shows the results of a statistical analysis performed (and plotted) with the MATLAB command *boxplot*. The distributions of the scale factors of the most influential parameters (G_Na_, G_K,ACh_, and v_NCX_) are markedly different in the two sub-groups (Fig. 4C). This simple analysis shows that the median values are different with a significance level of 0.05 (Fig. 4C and Table 2). Instead, there are no differences between the median values for G_Kur_, despite this parameter is shown to be important for P_EAD_ (see Fig. 4A). For completeness, we also report an example relative to a parameter that does not play a significant role in EAD formation in this context, such as G_Ks_, i.e., the maximal conductance of the slowly activating K^+^ current. The low regression coefficient associated to this parameter (Fig. 4A) makes P_EAD_ insensitive to perturbations in G_Ks_ (see flat relationship in Fig. 4B). Accordingly, also the distributions of the scale factors associated to G_Ks_ in the two sub-populations are not different (Fig. 4C and Table 2).

### Conclusions

Multivariable logistic regression analysis, applied here to investigate the factors responsible for arrhythmia development, is suitable for studying any process characterized by dichotomous outcome in both deterministic and stochastic models of myocyte electrophysiology and Ca^2+^ handling. For example, this predictive method has been applied successfully to analyze a stochastic model of Ca^2+^ sparks in the ventricular cell [2], and to investigate the drug-induced loss of automaticity in a deterministic model of the sinoatrial myocyte [8]. By using receiver operator characteristic analysis, Lee et *al*. [2] showed that logistic regression has the same predictive power of support vector machine, an established method for classifying binary data [24]. They also determined that this methodology is computationally more efficient than one-at-a-time parameter sensitivity analysis [2]. Therefore, application of logistic regression to populations of mathematical models is an efficient and effective technique that allows gaining novel biological insight and generating experimentally testable hypotheses, thereby improving our understanding of cardiac function in health and disease.

## Figures and Tables

**Table 1:** Definition of the perturbed model parameters.

Parameter	Definition
G_Na_	Conductance of the Na^+^ current
G_NaB_	Conductance of the background Na^+^ current
v_NKA_	Rate of the Na^+^/K^+^ pump
G_to_	Conductance of the transient outward K^+^ current
G_Kr_	Conductance of the rapidly activating K^+^ current
G_Ks_	Conductance of the slowly activating K^+^ current
G_Kur_	Conductance of the ultra-rapidly activating K^+^ current
G_Kp_	Conductance of the plateau K^+^ current
G_K1_	Conductance of the inward rectifier K^+^ current
G_K,ACh_	Conductance of the acetylcholine-sensitive K^+^ current
G_Cl(Ca)_	Conductance of the Ca^2+^-dependent Cl^−^ current
G_ClB_	Conductance of the background Cl^−^ current
G_Ca_	Conductance of the L-type Ca^2+^ current
G_CaB_	Conductance of the background Ca^2+^ current
v_PMCA_	Rate of the plasmalemmal Ca^2+^ pump
v_NCX_	Rate of the Na^+^/Ca^2+^ exchanger
v_SERCA_	Rate of the SERCA pump
v_RyR_	Rate of the SR Ca^2+^ release via ryanodine receptors
v_leak_	Rate of the SR Ca^2+^ leak via ryanodine receptors

**Table 2:** Properties of the scale factor distributions in the two sub-groups.

Parameter	EAD	Median		Notches (low/high)	Mean	Standard deviation
G_Na_	Yes	1.0466	*	1.0384/1.0548	1.0519	0.0948
	No	0.9439		0.9359/0.9519	0.9482	0.0815
G_K,ACh_	Yes	0.9567	*	0.9495/0.9639	0.9589	0.0803
	No	1.0672		1.0588/1.0756	1.0723	0.0906
v_NCX_	Yes	1.0193	*	1.0102/1.0284	1.0206	0.1007
	No	0.9667		0.9571/0.9763	0.9744	0.0948
G_Kur_	Yes	0.9982		0.9898/1.0067	0.9991	0.0962
	No	1.0101		1.0006/1.0195	1.0100	0.0990
G_Ks_	Yes	1.0002		0.9925/1.0079	1.0012	0.0902
	No	1.0067		0.9960/1.0174	1.0100	0.0989

*Asterisks indicate “strong evidence” (95% confidence) that the medians differ [25].
